# Durable Effect of Pyrotinib and Metronomic Vinorelbine in HER2-Positive Breast Cancer With Leptomeningeal Disease: A Case Report and Literature Review

**DOI:** 10.3389/fonc.2022.811919

**Published:** 2022-02-16

**Authors:** Yajing Chi, Mao Shang, Liang Xu, Heyi Gong, Rongjie Tao, Lihua Song, Baoxuan Zhang, Sha Yin, Binbin Cong, Huihui Li

**Affiliations:** ^1^ Department of Breast Medical Oncology, Shandong Cancer Hospital and Institute, Shandong First Medical University and Shandong Academy of Medical Sciences, Jinan, China; ^2^ School of Medicine, Nankai University, Tianjin, China; ^3^ Department of Oncology, Jinan Central Hospital, Cheeloo College of Medicine, Shandong University, Jinan, China; ^4^ Department of Radiology, Shandong Cancer Hospital and Institute, Shandong First Medical University and Shandong Academy of Medical Sciences, Jinan, China; ^5^ Department of Radiation Oncology, Shandong Cancer Hospital and Institute, Shandong First Medical University and Shandong Academy of Medical Sciences, Jinan, China; ^6^ Department of Neurosurgery, Shandong Cancer Hospital and Institute, Shandong First Medical University and Shandong Academy of Medical Sciences, Jinan, China; ^7^ Department of Breast Surgery, Shandong Cancer Hospital and Institute, Shandong First Medical University and Shandong Academy of Medical Sciences, Jinan, China

**Keywords:** case report, leptomeningeal metastases, HER2-positive breast cancer, pyrotinib, metronomic vinorelbine

## Abstract

Leptomeningeal metastases (LM) are rare and catastrophic for metastatic breast cancer (MBC). The prognosis of HER2-positive breast cancer (BC) with LM is extremely poor. There is no high-quality evidence of treatment regimens in HER2-positive BC with LM yet. Here, we present a case of LM in a 50-year-old woman with HER2-positive BC. Immunohistochemistry revealed invasive ductal carcinoma, estrogen receptor negative, progesterone receptor negative, HER2 3+, P53 positive 80%, and Ki-67 positive 35%. Reported for the first time, the patient was given pyrotinib-targeted therapy (400 mg, oral, every day), metronomic vinorelbine (40 mg, oral, three times a week), and intrathecal methotrexate (10 mg, infrequent and irregular use due to poor compliance) synchronously. The patient received and benefited from the treatment regimen for 16 months. And the quality of life, as self-reported, improved significantly. We also comprehensively summarized all the case reports, observational studies, and clinical trials related to HER2-positive BC with LM in the PubMed database and ClinicalTrials.gov. Intrathecal chemotherapy (methotrexate, cytarabine, thiotepa), intrathecal trastuzumab, whole-brain radiotherapy, and systemic therapy are commonly used treatment options according to a review of the literature and research. Pembrolizumab and trastuzumab deruxtecan (DS-8201) as novel drugs are promising in LM. Furthermore, trastuzumab emtansine (T-DM1) and tyrosine kinase inhibitors (TKIs) such as tucatinib and neratinib have exhibited good efficacy in HER2-positive BC with central nervous system (CNS) metastases and deserve further exploration. In our report, combining pyrotinib-targeted therapy with metronomic chemotherapy is a potential regimen, which has presented satisfactory therapeutic efficacy and also warrants additional investigation in HER2-positive BC with LM.

## Introduction

Breast cancer (BC) is the most widespread cancer and the primary tumor-related reason for death in women around the globe (1). Notably, accounting for 1% to 5% in BC, leptomeningeal metastases (LM) is closely linked to high mortality (2). The clinical symptoms of LM include headache, dizziness, nausea, vomiting, mental disorders, disturbances of consciousness, ataxia or weakness, etc. BC patients with LM have worse prognoses, compared to patients with metastases to other sites (3–5). The survival of untreated BC patients with LM is only about 1 month, and even after treatment, the average survival time seldom reach 8 months (6). Treatment choices mainly include radiotherapy, intrathecal chemotherapy, systemic treatment, and symptomatic care. There are still no available unified treatment protocols to date. Many treatment options exist, but combining or choosing among them remains difficult due to the limitations and of these therapies and their varying degree of tolerability by patients. Whether cerebrospinal radiotherapy can improve survival is still not clear enough. Intrathecal chemotherapy often exhibits potentially severe neurotoxicity, usually late in the course of treatment. In addition, systemic chemotherapy or targeted therapy has a limited ability to penetrate the blood-brain barrier.

Of all the subtypes, Human epidermal growth factor receptor 2 (HER2)-positive BC has a predilection for brain metastasis but is less likely to develop LM at the same time (7). Previous studies have reported that the frequency of HER2-positive BC among LM patients ranges between 14%-29% (8–12). Anti-HER2 targeted therapy is the preferred treatment regimen for patients with HER2-positive BC, and those with LM are no exception to this rule. Patients with LM, on the other hand, tend to have higher Eastern Cooperative Oncology Group (ECOG) scores (> 2), which might make traditional systemic treatments like chemotherapy and cerebrospinal radiotherapy difficult. There is also inadequate evidence to demonstrate the effectiveness of traditional macromolecular anti-HER2 drugs such as trastuzumab through the blood-brain barrier.

How to solve the aforementioned issues and enable patients to achieve long-term survival has a long way to go. Currently, most of the relevant research and clinical practice are still in their infancy. Herein, we present a case of HER2-positive BC with LM who was mainly treated with a combination of pyrotinib-targeted therapy and metronomic vinorelbine, resulting in a sustained therapeutic benefit for up to 16 months.

## Case Report

A 50-year old woman with no noteworthy medical history was diagnosed with left BC a decade ago and underwent a modified radical mastectomy: the left breast harbored an invasive ductal carcinoma. Additionally, 5 of 5 left intermuscular lymph nodes and 2 of 4 axillary lymph nodes were involved in metastasis. Immunohistochemistry found the presence of estrogen receptor negative, progesterone receptor negative, HER2 3+, P53 positive 80%, and Ki-67 positive 35%. Therewith, adjuvant chemotherapy consisting of 6 cycles of cyclophosphamide, epirubicin, and 5-fluorouracil was given to the patient, as well as radiotherapy to the left chest wall and supraclavicular area (50 Gy/25 fractions).

In November 2016, the patient developed metastasis to the left 10th rib, sternum, multiple lymph node metastases in the retroperitoneum, abdominal cavity, and mediastinum, and complicated with pleural effusion. However, the patient only received 6 cycles of docetaxel plus cyclophosphamide at the local hospital. After first-line treatment, the lesions could not be measured. And there was no evidence of progression until September 2017, when the patient had a single metastatic lesion of the right cerebellum. Over the following days, the patient got intensity-modulated radiotherapy (50Gy/10 fractions) targeting cerebellar metastasis, along with 4 cycles of gemcitabine plus capecitabine and trastuzumab simultaneously at the local hospital. The patient continued receiving trastuzumab for a year and was subsequently re-examined regularly with no signs of recurrence or new metastasis.

**Figure 1:**
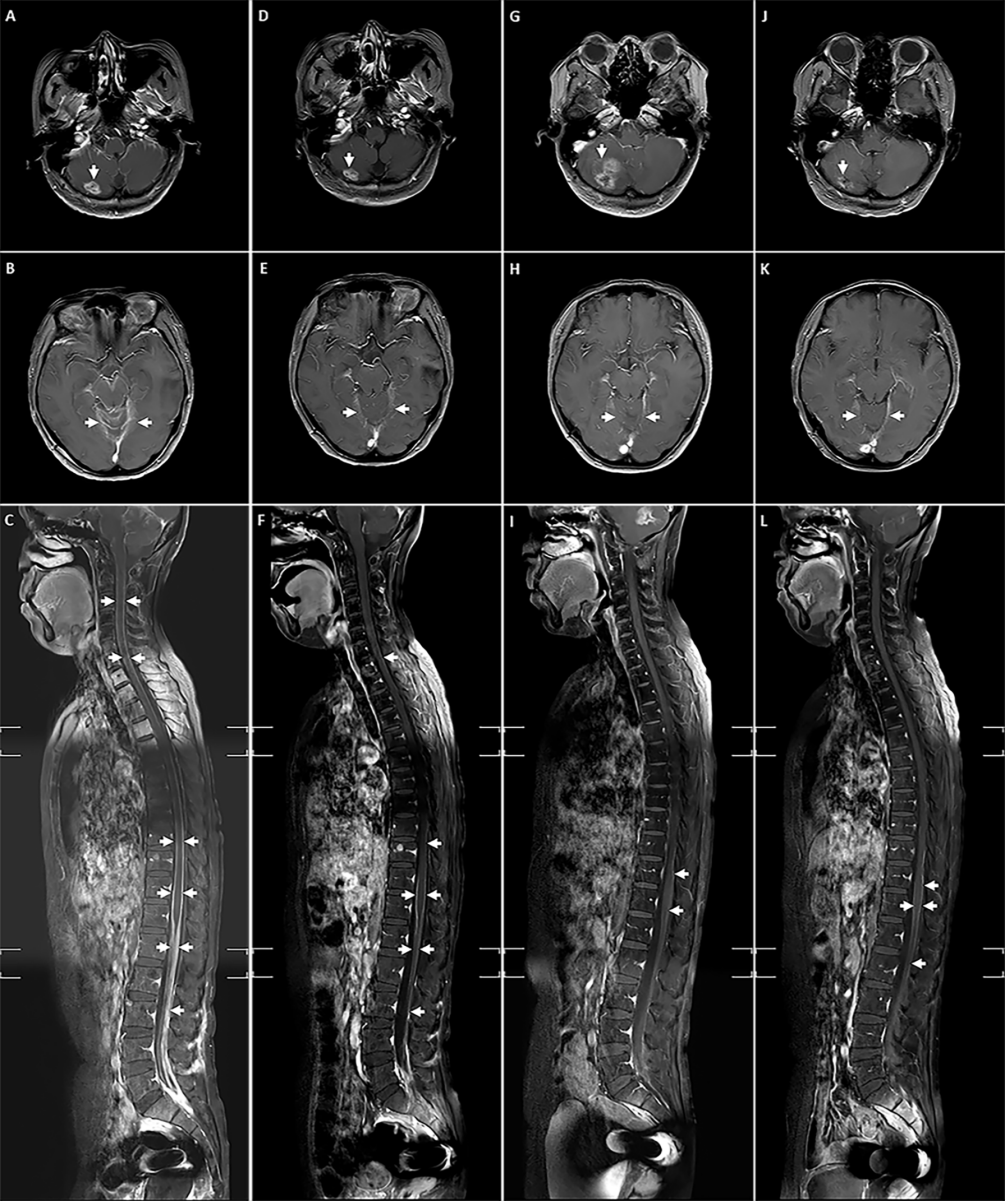
The lesions of right cerebellum metastasis, LM and spinal LM during treatment. **(A-C)** Before receiving pyrotinib combined with chemotherapy; **(D-F)** After 2 cycles of pyrotinib combining with chemotherapy; **(G-I)** Before the addition of WBRT; **(J-L)** After the addition of WBRT.

**Figure 2:**
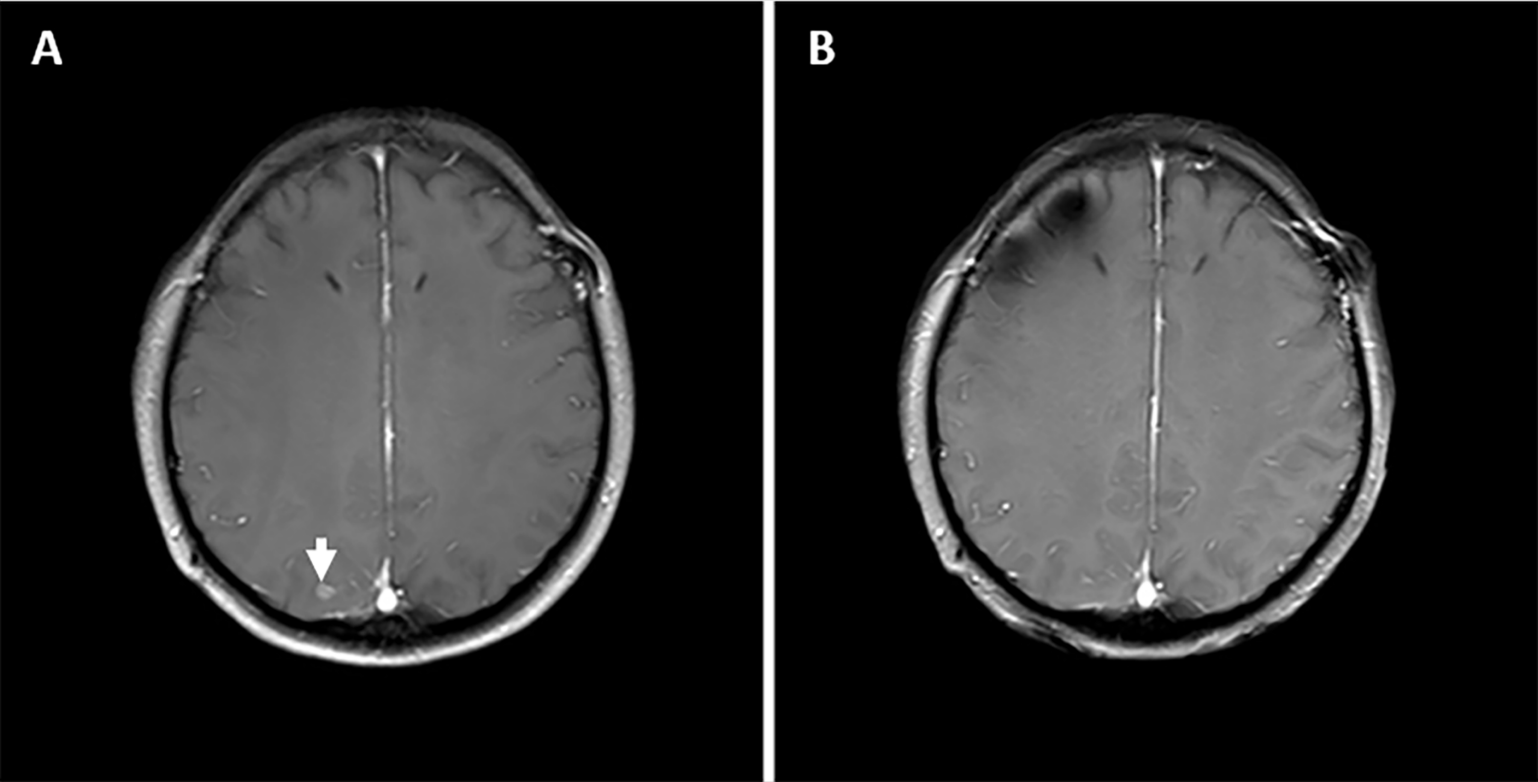
The status of right occipital lobe metastasis lesion during treatment. **(A)** Before the addition of WBRT. **(B)** After the addition of WBRT.

In December 2018, the patient had progressive onset of headache, nausea, vomiting, lower back pain, urinary retention, abnormal sensation in both lower extremities, constipation, and anal distension. The patient was subsequently admitted to our hospital in February 2019 for further treatment. A somewhat enlarged metastasis lesion to the right cerebellum was discovered on magnetic resonance imaging (MRI) after admission (**Figure 1A**). There were also abnormal strengthening signals in bilateral ventricular ependyma, meninges, spinal meninges, and cauda equina regions, which were identified and considered as LM (**Figures 1B, C**). A neurosurgeon performed right ventriculoperitoneal shunt and left ventricular Omaya reservoir implantation on the patient, followed by intrathecal injection of methotrexate (10 mg). A physician synchronously gave the patient pyrotinib-targeted therapy (400 mg, oral, daily), metronomic vinorelbine (40 mg, oral, three times a week), and intrathecal methotrexate (10 mg, occasional and irregular use due to poor compliance) since February 27, 2019. All symptoms were significantly relieved after 2 cycles of treatment, and the patient was able to urinate on her own, get out of bed, and walk with the assistance of her family members, and the quality of life improved drastically. Per the Common Terminology Criteria for Adverse Events (CTCAE) version 5.0, the patient developed Grade 1 diarrhea during treatment. Meanwhile, the synchronous MRI showed that the cerebella lesion had stabilized and that LM shrunk significantly. The overall evaluation of the curative effect pointed to partial response according to Response Evaluation Criteria in Solid Tumors (RECIST) version 1.1 (**Figures 1D–F**). In February 2020, a new metastasis appeared in the right occipital lobe, and the lesion in the right cerebellum was larger than before (**Figures 1G**, **2A**) but no LM progression (**Figures 1H, I**), so the patient received whole-brain radiotherapy (WBRT) (right occipital lobe lesion: 45Gy/10 fractions, whole-brain: 30Gy/10 fractions; considering that this was secondary radiotherapy to the brain, clinical target volume deducted the previous radiotherapy range of patient in 2017). After radiotherapy, the brain metastases shrunk significantly (**Figures 1J**, **2B**), and the patient continues to benefit from pyrotinib and metronomic vinorelbine. Notably, LM has always been in remission (**Figures 1K, L**). So far, the patient’s extracranial lesions have no signs of recurrence or metastasis, the central nervous system (CNS) metastases symptoms also have improved significantly beyond anyone’s expectations, and she has benefited from the current treatment regimen for 16 months.

## Methods

Literature and research were searched comprehensively in PubMed database and ClinicalTrials.gov by using the following keywords: leptomeningeal metastasis, leptomeningeal carcinomatosis, meningeal metastasis, meningeal neoplasms, meningeal carcinomatosis, neoplastic meningitis, carcinomatous meningitis, breast cancer, HER2-positive, case report, case series, observational study, prospective study, retrospective study, intervention study, clinical trial. Then, by combing the search results, we summarized the case reports of HER2-positive BC with LM, observational studies, and clinical trials related to LM in BC.

## Results

**Figure 3:**
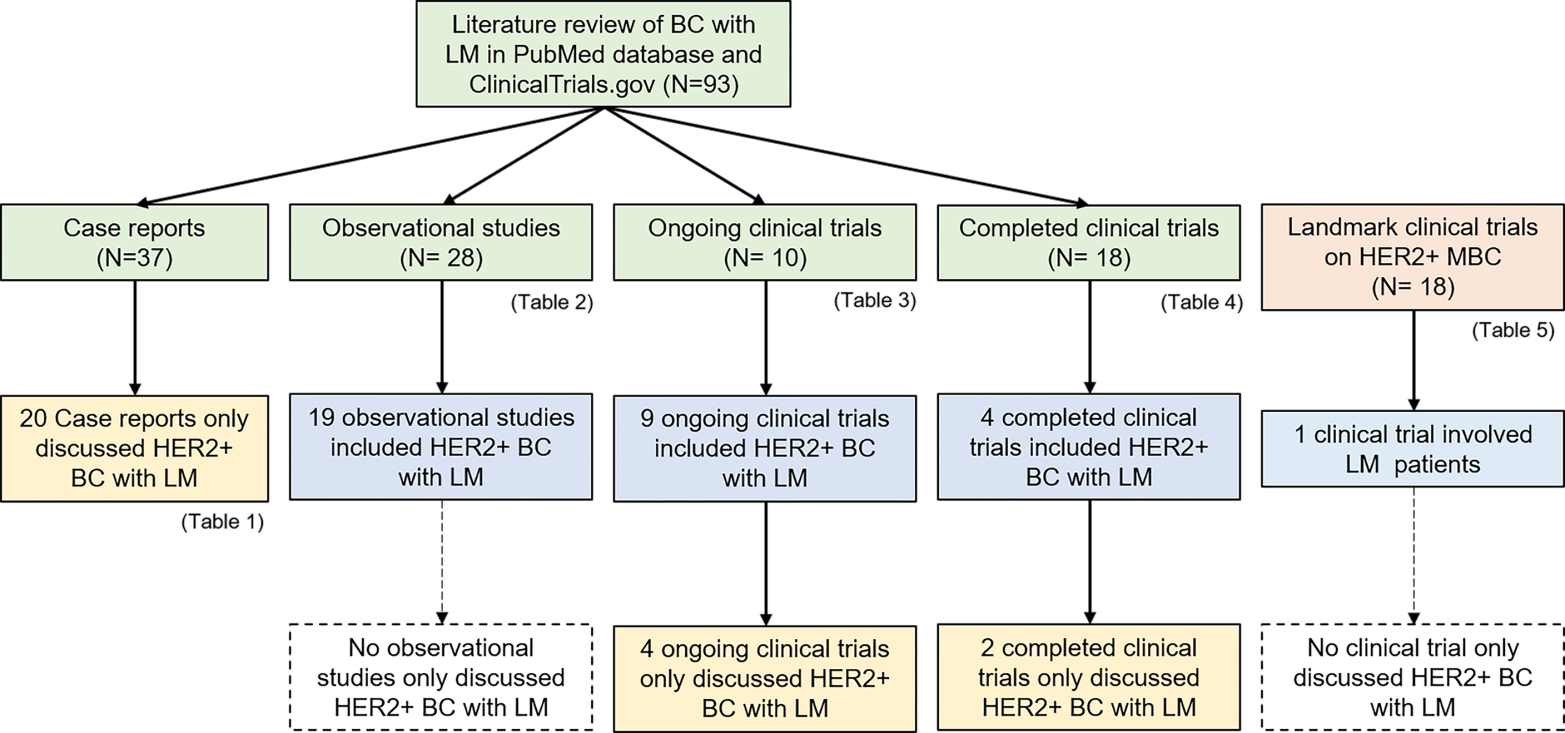
The summary of literature review for HER2-positive BC with LM.

Twenty case reports, 19 of 28 observational studies, 9 of 10 ongoing clinical trials, and 4 of 18 completed clinical trials in our review related to LM in BC included HER2-positive BC patients (**Figure 3**). Among these, all case reports, 4 ongoing clinical trials, and 2 completed clinical trials were specialized in HER2-positive BC with LM. Moreover, after an overview of 18 landmark clinical trials of HER2-positive MBC, only one clinical trial concerned LM. Literature review were summarized at following five tables.

### Case Reports of HER2-Positive BC With LM

A total of 25 cases of HER2-positive BC with LM were included in the review [**Table 1** (13–32)]. The median age of all patients was 43 years (range 31-75), with 9 cases of hormone receptor positive (47.3%), 12 cases of invasive ductal carcinoma (70.5%), and 19 cases (76.0%) with CNS parenchyma metastases. Median progression-free survival (mPFS) for all patients was 16.8 months. In terms of treatment, fourteen patients were treated with intrathecal chemotherapy (methotrexate 92.8%, cytarabine 28.5%, thiotepa 14.2%), about half of patients received intrathecal trastuzumab, and 9 patients received both. Any form of anti-HER2-targeted therapy, radiotherapy, and chemotherapy was given to 19, 12, and 21 patients, respectively.

**Table 1:** Summary of HER2-positive breast cancer with leptomeningeal metastases in case reports.

Ref	Year	Age	ER	^a^PR	Histopathological diagnosis	Complicated with other CNS metastases	Major treatment since diagnosis of LM	Best response (LM)	Survival (months)	Other treatment since diagnosis of LM
Current	2020	50	–	–	IDC	+	i.t. MTX, Pyr, p.o. Vin	PR	16	i.v. Tras, WBRT
García et al. (13)	2020	34	+	+	IDC	–	i.t. Tras, i.t. MTX, i.t. Ara-C	CCR	31	Lap, Let
Ricciardi et al. (14)	2018	46	–	–	IDC	+	WBRT, T-DM1	CR	>13	NA
Ji et al. (15)	2018	63	–	–	DCS	+	Erl	PR	>9	NA
Kordbacheh et al. (16)	2016	52	+	+	IDC	–	i.t. Tras, i.t. MTX	CR	>7.4	RT, i.v. Tras +Per
Park et al. (17)	2015	47	+	+	IDC	–	SRS, i.t. Tras, i.t. MTX	CR	20	i.v. Tras
	2015	42	–	–	IDC	+	i.t.Tras, i.v. Pac, i.v. Tras, WBRT	PR	>29	NA
Vincent et al. (18)	2013	45	–	–	IPDC	+	Cap, Bev	PR	28	Lap, i.v. Tras
Martens et al. (19)	2013	31	+	–	IDC	+	i.t. Tras, i.t. MTX	PR	5	i.v. Pac, Lap
Hofer et al. (20)	2012	75	NA	NA	NA	+	i.t. + i.v. Tras, WBRT	CR	>4	NA
	2009	38	–	–	IDC	+	i.t. + i.v. Tras	PR	>38	Temo, Cis + Cap
Mego et al. (21)	2011	43	NA	NA	NA	+	WBRT, i.t. MTX, i.t. Ara-C, i.v. Tras	PR	13.5	NA
	2011	39	NA	NA	NA	–	WBRT, i.t. MTX, i.t. Ara-C	PR	7.5	NA
Oliveira et al. (22)	2011	40	+	–	IDC	+	i.t. Tras, i.t. MTX, Exe	PR	27	Cap, Cis, Eto, i.v. Tras
Ferrario et al. (23)	2009	31	–	–	IDC	+	i.t. Tras, i.t. MTX, i.t. Thi	PR	>24	i.v. Tras, i.v. PLD, i.v. Vin, i.v. Pac
Hoffmann et al. (24)	2009	43	NA	NA	NA	+	WBRT, i.v. Ara-C, Tem	PR	9.5	NA
	2009	42	NA	NA	NA	+	i.v. Ara-C, Tem	SD	16.8	Cap, Lap
Shigekawa et al. (25)	2009	44	NA	NA	IDC	+	Cap	PR	10	i.v. Tras, i.v. Vin
Mir et al. (26)	2008	55	+	NA	NA	+	i.t. Tras	SD	7	NA
Stemmler et al. (27)	2008	48	–	–	NA	+	i.t. MTX, i.t. Tras	PR	14	i.v. Tras, Gem, Cis
Ekenel et al. (28)	2007	54	+	+	LIDC	+	Cap	SD	12	i.t. MTX, WBRT
Platini et al. (29)	2006	36	+	+	MLC	–	i.t. Tras, i.t. Thi	CCR	21	i.v. Pac, i.v. Tras, i.v. LD
Stemmler et al. (30)	2006	39	–	–	NA	+	i.t. Tras	CCR	5.2	i.v.Tras, Cap, i.t. MTX
Hikino et al. (31)	2006	54	–	–	IDC	+	Cap, WBRT	CR	>12	NA
Ozdogan et al. (32)	2003	41	+	–	LC	–	Let	CR	16	WBRT, i.t. MTX, Cis, Eto, i.t. Ara-C

Ara-C, cytarabine; Bev, bevacizumab; Cap, capecitabine; CCR, CSF and clinical response; Cis, cisplatin; CR, complete remission; DCS, Ductal carcinoma in situ; ER, estrogen receptor; Erl, Erlotinib; Eto, etoposide; Exe, exemestane; Gem, gemcitabine; IDC, Invasive ductal carcinoma; IPDC, Invasive poorly differentiated carcinoma; i.t., intrathecal; i.v., intravenous; Lap, lapatinib; LC, lobular carcinoma; LD, liposomal doxorubicin; LIDC, lobular and infiltrative ductal carcinoma; Let, letrozole; MLC, Multifocal lobular carcinoma; MTX, methotrexate; NA, not available; Pac, paclitaxel; Per, pertuzumab; PLD, pegylated liposomal doxorubicin; p.o., per os; ^a^PR, progesterone receptor; PR, partial remission; Pyr, pyrotinib; Ref, reference; RT, radiotherapy; SD, stable disease; SRS, Stereotactic radiotherapy; T-DM1, trastuzumab emtansine; Tem, temozolomide; Thi, thiotepa; Tras, trastuzumab; Vin, vinorelbine; WBRT, whole brain radiotherapy.

### Observational Studies in BC With LM

Finally, 28 observational studies were reviewed based on the search criteria [**Table 2** (9–11, 33–57)], with patients treated for LM ranging in time from 1976 to 2018. There was no report specifically addressed the LM of HER2-positive BC. However, a subset of HER2-positive patients was included in 18 of 28 studies. Intrathecal chemotherapy (methotrexate, cytarabine, and thiotepa, etc), WBRT, and systemic therapy were the main treatment options in all studies, with some patients receiving surgical intervention. The efficacy of combination therapy was assessed in 26 studies, with median progression-free survival (mPFS) ranging from 1.6 to 4.2 months for all patients, median overall survival (mOS) ranging from 0.9 to 7.7 months for all patients, and 1.5 to 8.4 months for HER2-positive patients. Only one retrospective study observed the efficacy of WBRT with a mOS of 2.1 months for all patients and 5.3 months for HER2-positive patients. There was also only one case series that reviewed the efficacy of intravenous thiotepa alone in LM of BC, with mOS rate of 69% and 31%, respectively, at 6 months and 12 months.

**Table 2:** Summary of breast cancer with leptomeningeal metastases in observational studies.

Ref	Year	Pts (n)	HER2+	Median/Mean age (yrs)	Major treatment since diagnosis of LM (rate)	Other CNSM	Therapeutic response	mPFS (months)	mOS (months)
Okada et al. (33)	2010-2018	31	29%	58.0	WBRT (100%)	67.7%	NA	NA	All: 2.1, HER2+: 5.3
Chahal et al. (34)	2010-2013	13	38.5%	51	i.v. Thi (100%)	NA	ORR 31%	NA	mOS at 6 months: 69%
							DCR 54%		mOS at 12 months: 31%
Du et al. (35)	2008-2011	46	NA	All: 53	All: i.t. chemo (10.9%), WBRT/WBRT+SRS (45.7%), systemic treatment (56.5%)	All: 54.3%	NA	NA	All:4.4, BC: 4.0
		(BC: 5)							
Kim et al. (36)	2007-2017	58	NA	All: 51	All: i.t. MTX/MTX + Ara-C (74.1%), RT (29.3%), systemic chemo (41.4%)	NA	NA	NA	All: 2.4
		(BC: 23)							
Fusco et al. (37)	NA	27	NA	All: 49	BC: i.t. liposomal Ara-C (100%), WBRT (6.6%), systemic chemo (33.3%)	NA	All: ORR 33.3%	NA	All:1.4, BC: 0.9
		(BC: 15)					DCR 62.9%		
							BC: ORR 33.3%		
							DCR 40%		
Le Rhun et al. (38)	2007-2011	103	28.42%	53	i.t. liposomal Ara-C in 1^st^ line (100%), i.t. Thi 2^nd^ line (23%), i.t. MTX in 3^rd^ line (6%), RT (17%), systemic treatment (58%)	51.46%	ORR 57%	1^st^ line: 2.1	3.8
								2^nd^ line: 2.8	
								3^rd^ line: 4.2	
Jaeckle et al. (39)	2006-2016	31	13%	58	i.t. TOPO (100%), RT (84%), systemic therapy (77%)	NA	ORR 13%	2.5	6.9
							DCR 68%		
Kingston et al. (40)	2004-2014	182	26.4%	52.5	i.t. chemo (7.7%), i.t. Tras (1.1%), whole or partial brain RT (34.1%), systemic therapy (24.7%),	50%	NA	3.9	5.4
Cochereau et al. (41)	2003-2015	41	15%	59	i.t. MTX (100%), RT 20%, systemic chemotherapy (68%)	NA	ORR 54%	NA	4.0
							DCR 61%		
Quigley et al. (42)	2003-2010	88	All: 55%	All: 56.9	LM: i.t chemo (6.5%), spinal RT (45.2%), i.t chemo + spinal RT (3.2%)	100%	NA	NA	All:9.7, LM:1.8
		(LM:31)							
de Azevedo et al. (9)	2003-2009	60	15%	46	i.t. MTX (68.3%), RT (36.7%), systemic treatment (21.6%)	41.6%	NA	NA	All: 3.3
									mOS at 12 months: 29.6%
Lara-Medina et al. (43)	2003-2007	49	20%	42.4	i.t. MTX (59%), surgical procedures (20%), RT (65%), systemic chemo (53%)	49%	NA	NA	All: 1.75, HER2+: 1.5
Griguolo et al. (44)	2002-2017	153	20.9%	58	i.t. MTX/liposomal Ara-C/Tras/Thi (67.3%), surgical derivation (9.8%), WBRT/spinal RT/other RT (27.5%), systemic treatment (71.9%), Anti-HER2 therapy in HER2+ BC (68.8%)	43.1%	NA	NA	All: 3.9, HER2+: 8.4
Comte et al. (45)	2000-2012	66	16%	54	i.t. Thi in 1^st^ line (100%), i.t. chemo + RT (23%), i.t. MTX in 1^st^ line+i.t. Thi in 2^nd^ line (83.3%)	NA	NA	MTX:3.7	All: 4.5
								Thi in 1^st^ line:1.6	Thi in 1^st^ line: 3.3
Gauthier et al. (46)	2000-2007	91	10%	53	i.t. MTX (87.9%), i.t. Thi (36%), RT (29%), systemic chemo (78%)	38%	ORR 72%	NA	4.5
Rudnicka et al. (47)	2000-2005	67	NA	49	i.t. MTX (85%), i.v. chemo (61%), WBRT (49%), spinal cord RT (15%), combined modality (40%)	33%	ORR 76%	NA	4
Niwińska et al. (48)	1999-2015	187	19%	49	i.t. MTX/liposomal Ara-C (68%), RT (56%), systemic therapy (56%)	36%	NA	NA	4.2
Niwińska et al. (49)	1999-2011	149	22%	49	i.t. MTX (54.4%), i.t. liposomal Ara-C (10.1%), RT (61.7%), systemic therapy (51.7%)	37%	ORR 63%	NA	4.2
Clatot et al. (50)	1999-2008	24	29%	49	i.t. MTX (100%), RT (46%), systemic chemo (46%)	25%	NA	NA	5
Morikawa et al. (10)	1998-2013	318	26%	54	i.t./i.v. therapy (14%), RT (64%), i.v. MTX (20%), VP shunt (19%)	66%	NA	NA	All: 3.5, HER2+: 5.2
Kosmas et al. (51)	1996-2000	310	LM:28%	LM: 54	LM: i.t. MTX+WBRT (71.4%), i.t. MTX+RT to lumbar spine (14.3%)	NA	All: ORR 28.6%	NA	LM: 3.6
		(LM: 7)					DCR 57.2%		
							LM: ORR 28.6%		
Yust-Katz et al. (52)	1995-2011	103	47.4%	49.2	i.t. chemo (MTX 17%, TOPO 22%, Ara-C 24%, Multiple 9%), WBRT (52.5%), spinal RT (19%), systemic chemo (36.4%)	46.1%	NA	NA	4.2
Lee et al. (11)	1995-2008	68	27.9%	All: 46	All: i.t. chemo (14.7%), WBRT (14.7%), combination modality (61.8%)	100%	NA	NA	All: 4.1, HER2+: 5.9
				HER2+:47	HER2+ (n=18): i.t. chemo (11.1%), WBRT (11.1%), combination modality (77.8%)				
Chamberlain et al. (53)	1990-2007	60	NA	BC: 59.5	BC: i.t. chemo (100%), RT (90%), systemic chemo (60%)	NA	NA	NA	All: 1.5, BC: 3
		(BC: 20)							
Yu et al. (54)	1990-1999	8	NA	51.5	i.t. MTX and/or immunotherapy (37.5%), WBRT (100%)	NA	NA	NA	WBRT alone: 4.0
									WBRT + i.t. therapy: 5.37
Chamberlain et al. (55)	1986-1995	32	NA	49	i.t. MTX in 1^st^ line (65.3%), i.t. Ara-C 2^nd^ line (44.9%), i.t. Thi in 3^rd^ line (22.4%), RT (42.9%)	NA	ORR: MTX 43.8%	NA	7.5
							Ara-C 36.4%		
							Thi 27.3%		
Jayson et al. (56)	1979-1992	35	NA	45	i.t. MTX (31.4%), i.t. Thi (2.9%), i.t. Ara-C (2.9%),	NA	DCR 63%	NA	2.57
					i.t. MTX+RT (8.6%), RT (17.1%)				
					i.v. MTX (28.6%), other systemic chemo (14.3%)				
Strady et al. (57)	1976-1996	41	NA	All: 57	BC: i.t. MTX (66.7%), systemic chemo (77.8%)	All: 39%	NA	NA	BC: 1.1
		(BC: 18)		BC: 56		BC: 0%			

Ara-C, cytarabine; BC, breast cancer; CNSM, central nervous system metastases; DCR, disease control rate; i.t., intrathecal; i.v., intravenous; LM, leptomeningeal metastases; MTX, methotrexate; mOS, median overall survival; mPFS, median progression-free survival; NA, not available; ORR, objective response rate; Pts, patients; Ref, reference; SRS, Stereotactic radiotherapy; RT, radiotherapy; Tras, trastuzumab; Thi, thiotepa; TOPO, topotecan; VP, ventriculoperitoneal; WBRT, whole brain radiotherapy.

### Clinical Trials in Solid Tumors With LM Including BC

As far as we searched on ClinicalTrials. gov, until now, there were 10 ongoing clinical trials related to LM in BC [**Table 3** (58–67)]. Except for the ANGLeD study, the others were single-arm and phase I/II trials focused on HER2-positive/low expressing BC with LM. Novel anti-HER2-targeted drugs such as trastuzumab deruxtecan (DS-8201) and tucatinib are also being investigated. Currently, most of the clinical trials involving BC with LM that have obtained advances are designed as basket trials [**Table 4** (68–85)].

**Table 3:** Summary of ongoing clinical trials of breast cancer with leptomeningeal metastases registered on clinicaltrials.gov.

NCT No./Study	Phase	Molecular status	Pts (n)	Study design
NCT03501979/TBCRC049 (58)	II	HER2+	30	Tuc + i.v. Tras + Cap
NCT04588545 (59)	I/II	HER2+	39	RT followed by i.t. Tras + i.t. Per
NCT04420598/DEBBRAH (60)	II	HER2+/low expressing status	39	i.v. DS-8201
NCT03696030 (61)	I	HER2+	39	i.v. HER2-Targeted Chimeric Antigen Receptor (HER2-CAR) T Cells
NCT02650752 (62)	I	HER2+	11	Lap + Cap
NCT03613181/ANGLeD (63)	III	HER2-	150	ANG1005 *vs*. Physician’s Best Choice
NCT03661424 (64)	I	Any	16	i.v. HER2 Bi-armed activated T-cells (BATs)
NCT02422641 (65)	II	Any	16	i.v. high-dose MTX
NCT01818713 (66)	II	Any	6	Glutathione pegylated liposomal doxorubicin hydrochloride formulation (2B3-101)
NCT00992602 (67)	II	Any	3	i.v. high-dose MTX + i.t. liposomal Ara-C

Ara-C, cytarabine; Cap, capecitabine; DS-8201, trastuzumab deruxtecan; i.t., intrathecal; i.v., intravenous; Lap, lapatinib; MTX, methotrexate; Pts, patients; Per, pertuzumab; Ref, reference; RT, radiotherapy; Tras, trastuzumab; Tuc, tucatinib.

**Table 4:** Summary of clinical trials in solid tumors (including breast cancer) with leptomeningeal metastases.

Ref	NCT No./Study	Basket trial	Year	Phase	Pts(n)	Study design	HER2 + in BC	Median/Mean age (years)	Survival (months)
Le Rhun et al. (68)	NCT01645839/DEPOSEIN	No	2020	III	74	i.t. liposomal Ara-C + systemic therapy *vs*. systemic chemo alone	15%	57	mPFS 3.8 *vs*. 2.2 (P = 0.04) mOS 7.3 *vs*. 4.0 (P = 0.51)
Jarushka Naidoo et al. (69)	NCT03091478	Yes	2020	II	13 (BC: 5)	i.v. Pembrolizumab	NA	58	NA (ORR 61.5%)
Malani et al. (70)	NCT01325207	No	2020	I/II	14	i.t. Tras	100%	NA	NA
Pan et al. (71)	NCT03507244	Yes	2020	I/II	34 (BC: 4)	i.t. Pem + RT	NA	NA	All: mNPFS 3.5, mOS 5.5
Bonneau et al. (72)	NCT01373710	No	2018	I	16	i.t. Tras	100%	57	mOS 7.3
Wu et al. (73)	NCT01281696	No	2015	III	8	i.v. Bev + Eto + Cis	25%	55	mNPFS 4.7, mOS 4.7
Ursu et al. (74)	NA	Yes	2015	I	29 (BC: 3)	i.h. + i.t. CpG-28	NA	56	mPFS 1.75, mOS 3.75
Bernardi et al. (75)	NCT00074607	Yes	2008	I	10 (BC: 3)	i.t. Gem	NA	25	NA (CR: None; SD: 3 patients)
Chamberlain et al. (76)	NA	Yes	2006	II	27 (BC: 5)	i.t. Eto	NA	55	mNPFS 5
Boogerd et al. (77)	NA	No	2004	I	35	i.t. chemo *vs*. non i.t. chemo	NA	i.t. chemo: 49.6	mPFS 5.75 *vs*. 6
								control: 57.9	mOS 4.58 *vs*. 7.58 (P=0.32)
Blaney et al. (78)	NA	Yes	2003	I	23 (BC: 2)	i.t. TOPO	NA	12	NA
Orlando et al. (79)	NA	No	2002	I	13	i.t. Thi + MTX + Ara-C	NA	45	mOS 2.1
Chamberlain (80)	NA	Yes	2002	II	22 (BC: 3)	i.t. Alpha-interferon	NA	56	mOS 4.5
Jaeckle et al. (81)	NA	Yes	2002	NA	110 (BC: 38)	i.t. Ara-C	NA	50	mNPFS 1.83, mOS 3.17
Jaeckle et al. (82)	NA	No	2001	I	56	i.t. liposomal Ara-C	NA	50	mPFS 1.63, mOS 2.0
Glantz et al. (83)	NA	Yes	1999	NA	61 (BC: 22)	i.t. liposomal Ara-C *vs*. i.t. MTX	NA	49	mNPFS 1.93 *vs*. 1.0 (P = 0.007)
									mOS 3.5 *vs*. 2.6 (P = 0.15)
Grossman et al. (84)	NA	Yes	1993	NA	52 (BC: 25)	i.t. MTX *vs*. i.t. Thi	NA	NA	mOS 3.98 *vs*. 3.53
Hitchins et al. (85)	NA	Yes	1987	NA	44 (BC:11)	i.t. MTX/MTX + Ara-C	NA	55	All: mOS 2

Ara-C, cytarabine; BC, breast cancer; Bev, bevacizumab; Cis, cisplatin; CLC, colon cancer; CNS, central nervous system; CR, complete response; Eto, etoposide; Gem, gemcitabine; i.h., hypodermic injection; i.t., intrathecal; i.v., intravenous; LC, lung cancer; MTX, methotrexate; mOS, median overall survival; mPFS, median progression-free survival; mNPFS, median neurologic progression-free survival; NA, not available; NSCLC, non-small cell lung cancer; ORR, objective response rate; Pts, patients; Pem, pemetrexed; Ref, reference; RT, radiotherapy; SCLC, small cell lung cancer; SD, stable disease; Tras, trastuzumab; Thi, thiotepa; TOPO, topotecan.

In **Table 4**, 16 of 18 studies explored the efficacy of intrathecal treatment in LM, with mOS ranging from 2.0 to 7.3 months. The mOS of HER2-positive patients who received intrathecal trastuzumab could also reach 7.3 months in a phase I clinical trial. Two randomized controlled trials compared intrathecal chemotherapy with non-intrathecal chemotherapy in BC: a phase III study showed that intrathecal chemotherapy provided better survival compared to the control group (mPFS 3.8 *vs*. 2.2 months, *p* = .04; mOS 7.3 *vs*. 4.0 months, *p* = .51), but worse survival (mOS 4.58 *vs*. 7.58 months, *p* = .32) with treatment-related neurotoxicity in another study. According to a study in 1999, intrathecal cytarabine enhanced median intracranial PFS (1.93 *vs*. 1.0 months, *p* = .007) in comparison to methotrexate. Intravenous pembrolizumab achieved an objective response rate (ORR) of 61.5% in a recent phase II clinical trial of only 13 patients with LM in solid tumors (including BC).

### Landmark Clinical Trials of HER2-Positive MBC

Eighteen landmark clinical trials for HER2-positive MBC were included in the literature review [**Table 5** (86–110)]. Seven studies excluded patients with LM, and 10 studies did not clarify. Only the TBCRC 022 study allowed the inclusion of patients with LM while investigating the efficacy of neratinib plus capecitabine in HER2-positive BC with brain metastases (BM), and three patients with LM had progressive disease, partial response, and stable disease, respectively. The CLEOPATRA, PUFFIN, ALTERNATIVE, and PHOEBE study completely excluded CNS metastases, whereas the CLEOPATRA study analyzed the occurrence of CNS metastases: the pertuzumab group took longer time than the placebo group to the onset CNS metastases (15.0 *vs*. 11.9 months, *p* = .0049), and for patients with CNS metastases, the pertuzumab group seemed to have a longer survival (34.4 *vs*. 26.3 months, *p* = .1139). Although the BOLERO-3, PERUSE, and SOPHIA study included CNS metastases, no results have been reported for CNS metastases. In the DESTINY-Breast 01 study, DS-8201 reached a mPFS of 18.1 months in patients with BM. The mOS of patients with CNS metastasis treated by trastuzumab emtansine (T-DM1) in the EMILIA and TH3RESA study was 26.8 and 17.3 months, respectively, which was better than those of the control group. The LANDSCAPE study showed that the mPFS of patients with HER2-positive BM treated with lapatinib plus capecitabine was 5.5 months with an ORR of 65.9%. In the BM subgroup of the HER2CLIMB study, the mPFS of tucatinib group was longer than the placebo group (7.6 *vs*. 5.4 months, *p* <.001). In the NEfERT-T study, the neratinib group had a lower incidence of CNS recurrences than the trastuzumab group (8.3% *vs*. 17.3%, *p* = .002), and also a lower overall cumulative incidence of intervention for CNS disease than the lapatinib group (22.8% *vs*. 29.2%, *p* = .043) in NALA study.

**Table 5:** Summary of landmark clinical trials of HER2-positive metastatic breast cancer.

Study (NCT No.)	Phase	Pts (n)	Study design (n)	Therapy line	CNSM (n)	BM (n)	LM (n)	Survival (months)
EMILIA (86–88) (NCT00829166)	III	991	T-DM1 (495) *vs*. Lap + Cap (496)	2^nd^	+ (90)	+	NA	All: mPFS 9.6 *vs*. 6.4 (P<0.001), mOS 29.9 *vs*. 25.9 CNS:
								CNSM: mPFS 5.9 *vs*. 5.7 (P=0.9998), mOS 26.8 *vs*. 12.9 (P=0.0081)
CLEOPATRA (89–91) (NCT00567190)	III	808	Per + Tras + Doc (402) *vs*. Pla + Tras + Doc (406)	1^st^	–	–	–	All: mPFS 18.7 *vs*. 12.4, mOS 57.1 *vs*. 40.8
								CNSM: Median time to onset of CNSM 15.0 *vs*. 11.9 (P = 0.0049)
								mOS 34.4 *vs*. 26.3 (P=0.1139)
LANDSCAPE (92) (NCT00967031)	II	45	Lap + Cap	1^st^	+	+ (45)	NA	All/BM: mPFS 5.5, ORR 65.9%
TH3RESA (93, 94) (NCT01419197)	III	602	T-DM1(404) *vs*. Physician’s Choice (198)	median line: 4^th^	+	+ (72)	NA	All: mPFS 6.2 *vs*. 3.3 (P<0.0001), mOS 22.7 *vs*. 15.8 (P=0.0007)
								BM: mPFS 5.8 *vs*. 2.9, mOS 17.3 *vs*. 12.6
BOLERO-3 (95) (NCT01007942)	III	569	Eve+ Vin + Tras (284) *vs*. Pla + Vin + Tras (285)	2^nd^	+ (27)	–	–	All: mPFS 7.00 *vs*. 5.78 (P=0.0067)
NEfERT-T (96) (NCT00915018)	III	479	Ner + Pac (242) *vs*. Tras + Pac (237)	1^st^	+ (18)	+	NA	All: mPFS 12.9 *vs*. 12.9 (P=0.89)
								Incidence of CNS recurrences: 8.3% *vs*. 17.3% (P=0.002)
MARIANNE (97) (NCT01120184)	III	1095	Tras + Tax (365) *vs*. T-DM1 + Pla (367) *vs*. T-DM1 + Per (363)	1^st^	NA	NA	NA	All: mPFS 13.7 *vs*. 12.9 (P=0.31) *vs*. 15.2 (P=0.14)
ALTERNATIVE (98) (NCT01160211)	III	355	Lap + Tras + AI (120) *vs*. Tras + AI (117) *vs*. Lap + AI (118)	1^st^/2^nd^	–	–	–	All: mPFS 11 *vs*. 5.7 (P=0.0064) *vs*. 8.3 (P=0.0361)
								mOS 46.0 (P=0.07) *vs*. 40.0 *vs*. 45.1 (P=0.440)
PHENIX (99) (NCT02973737)	III	279	Pyr + Cap (185) *vs*. Pla + Cap (94)	2^nd^	+	+ (31)	NA	All: mPFS 11.1 *vs*. 4.1 (P<0.001)
								BM: mPFS 4.2 *vs*. 6.9 (P=0.011)
PERUSE (100) (NCT01572038)	III	1436	Per + Tras + Tax (Doc 775, Pac 589, Nab-Pac 65)	1^st^	+	+	NA	All: mPFS 20.6 (Doc 19.6, Pac 23.0, Nab-Pac 18.1)
TBCRC 022 (101) (NCT01494662)	II	49	Ner + Cap (No prior Lap: 37, Prior Lap: 12)	Any	+	+ (49)	+ (3)	ALL/BM: No prior Lap: mPFS 5.5, mOS 13.3, Prior Lap: mPFS 3.1, mOS 15.1
								LM:3 patients had PD, PR, SD, respectively
KAMILLA (102, 103) (NCT01702571)	III	2003	T-DM1	Any	+	+ (398)	NA	Cohort1: mPFS 6.9, mOS 27.2
								BM: mPFS 5.5, mOS 18.9
NALA (104) (NCT01808573)	III	621	Ner + Cap (307) *vs*. Lap + Cap (314)	≥3^rd^	+	+ (101)	–	All: Mean PFS: 8.8 *vs*. 6.6 (P=0.0003)
								BM: mPFS 5.5, mOS 18.9
PUFFIN (105) (NCT00567190)	III	243	Per + Tras + Doc (122) *vs*. Pla + Tras + Doc (121)	1^st^	–	–	–	All: mPFS 14.5 *vs*. 12.4, mOS 57.1 *vs*. 40.8
DESTINY-Breast 01 (106, 107) (NCT03248492)	II	184	DS-8201	median line: 6^th^	+	+ (24)	NA	All: mPFS 19.4, mOS 24.6
								BM: mPFS 18.1
HER2CLIMB (108) (NCT02614794)	II	612	Tuc + Tras+ Cap (410) *vs*. Pla + Tras+ Cap (202)	median line: 3^rd^	+	+ (219)	–	All: mPFS 7.8 *vs*. 5.6 (P<0.001), mOS 21.9 *vs*. 17.4 (P=0.005)
								BM: mPFS 7.6 *vs*. 5.4 (P<0.001)
PHOEBE (109) (NCT03080805)	III	266	Pyr + Cap (134) *vs*. Lap + Cap (132)	≤3	–	–	–	All: mPFS 12.5 *vs*. 6.8 (P<0.0001)
SOPHIA (110)(NCT02492711)	III	536	Mar + Chemo (266) *vs*. Tras + Chemo (270)	≥2	+	+ (71)	NA	All: mPFS 5.8 *vs*. 4.9 (P=0.03), mOS 21.6 *vs*. 19.8 (P=0.33)

AI, aromatase inhibitor; BM, brain metastases; Cap, capecitabine; CNS, central nervous system; CNSM, central nervous system metastases; Doc, Docetaxel; DS-8201, Trastuzumab deruxtecan; Eve, everolimus; Lap, lapatinib; LM, leptomeningeal metastases; Mar, Margetuximab; mOS, median overall survival; mPFS, median progression-free survival; NA, not available; Nab-Pac, nab-paclitaxel; Ner, neratinib; Pac, paclitaxel; PD, Progressive disease; Per, pertuzumab; Pla, placebo; Pts, patients; PR, partial response; Pyr, pyrotinib; Ref, reference; Tax, taxane; T-DM1, trastuzumab emtansine; Tuc, tucatinib; Tem, temozolomide; SD, stable disease; Vin, vinorelbine.

## Discussion

Treatment for patients with LM should be based on multidisciplinary cooperation. According to the EANO-ESMO Clinical Practice Guidelines, patients with LM can choose intrathecal chemotherapy, radiotherapy, and systemic chemotherapy as the primary treatment (111). The above treatment regimens were also the predominant therapeutic options in the case reports and observational studies we reviewed (**Tables 1**, **2**). However, there was rare large and randomized controlled clinical trial yet to demonstrate that the above therapeutic choices are efficient for BC with LM. Only two randomized controlled trials compared intrathecal chemotherapy with non-intrathecal chemotherapy in BC but came to opposite results. Furthermore, the majority of clinical trials only explored the efficacy of intrathecal treatment of LM with merely 7.3 months of optimal mOS (**Table 4**). In reviewed literature, 48% of the case reports and 26 of 28 observational studies of patients received radiotherapy, and most of the patients received systemic treatment. These studies, to various degrees, mirrored the effectiveness of radiotherapy and systemic treatment in patients with LM. Taking into account the poor physical condition of the current case, the radiologists reluctantly gave up the cerebrospinal radiotherapy that had been planned for her.

Anti-HER2 monoclonal antibody, antibody-drug conjugates, or tyrosine kinase inhibitors (TKIs) can be selected as targeted therapy regimens in HER2-positive MBC with LM. We noted that the therapeutic outcomes were satisfactory in several case reports where intrathecal trastuzumab was used (**Table 1**). There was no observational study discussed solely for LM of HER2-positive BC (**Table 2**), indicating that there is still a dearth of attention on LM of HER2-positive BC, which necessitates further efforts and exploration, also may be due to premature cases inclusion (during a time when the idea of HER2 was unknown) and the difficulty in case collection. Previous landmark clinical trials for patients with HER2-positive MBC either excluded patients with LM or did not specify whether patients with LM were included (**Table 5**). And the number of patients with LM was too small in TBCRC 022 study to draw firm conclusion. However, the CLEOPATRA study has shown that pertuzumab + trastuzumab + docetaxel delayed the occurrence of BM, and patients with BM tend to have a better survival in this group. Gratifyingly, the DESTINY-Breast 01, EMILIA, LANDSCAPE, HER2CLIMB, and NALA study respectively confirmed that DS-8201, T-DM1, lapatinib, tucatinib, and neratinib are effective in HER2-positive BC with CNS metastasis. And a recent phase II clinical trial enrolled thirteen patients (tumor agnostic) with LM including BC showed a benefit of pembrolizumab, which suggests that immunotherapy also has a potential in the treatment for LM (**Table 4**). Unfortunately, T-DM1, DS-8201, pertuzumab, tucatinib, neratinib, and pembrolizumab were not yet available in mainland China at the time our patient was diagnosed as LM. Moreover, patients with LM are generally in poor condition, making high-intensity treatment regimens difficult to accept. And intrathecal trastuzumab is an off-label medication used in China. Hence, newer, better, safer, and more effective treatment options for HER2-positive BC with LM are urgently needed.

Pyrotinib is a novel irreversible epidermal growth factor receptor (EGFR)/HER2 dual TKI. Currently, several clinical trials, such as the PHOEBE study (NCT03080805) and the PHENIX study (NCT02973737) which were presented orally by Chinese scholars at the ASCO meeting, have confirmed that pyrotinib is effective and tolerable in HER2-positive MBC. The ORR (67.2% *vs*. 51.5%) and mPFS (12.5 *vs*. 6.9 months, *p* <.0001) in the capecitabine plus pyrotinib group were superior to lapatinib plus capecitabine group in patients with HER2-positive MBC, who had been given taxanes, anthracycline, and/or trastuzumab and recruited by PHOEBE study. Compared with the combination of placebo and capecitabine, pyrotinib plus capecitabine was demonstrated to significantly prolong the mPFS (11.1 *vs*. 4.1 months, *p* <.001) in patients with HER2-positive MBC previously treated with trastuzumab and taxanes in the PHENIX study. The subgroup analysis indicated that pyrotinib plus capecitabine prolonged mPFS in patients with BM at baseline by 2.7 months compared with the placebo group (6.9 *vs*. 4.2 months, *p* = .011) with fewer BM progression (73.3% *vs*. 87.5%), and longer median time to BM progression (168 *vs*. 127 days). Among patients without BM, the pyrotinib group had a lower incidence of BM than the placebo group (1.2% *vs*. 3.6%), and a longer median time to developing new BM (397.5 *vs*. 132.0 days). Moreover, preliminary results from an ongoing study (NCT03691051) in the Chinese population presented at the 2020 ESMO meeting showed that combining pyrotinib with capecitabine resulted in an ORR of 76.9% in HER2-positive MBC with BM (112). In terms of mechanism, the molecular weight of pyrotinib is 815.22 Da (<1000 Da), which could allow pyrotinib to penetrate past the cell membrane and operate directly on intracellular targets. Hence, the effectiveness in CNS metastases may be due to the possibility that pyrotinib may penetrate the blood-brain barrier as a small-molecule anti-HER2 TKI like lapatinib (113). And different from lapatinib, pyrotinib can irreversibly and simultaneously inhibit HER1, HER2, and HER4, thereby blocking RAS/RAF/MEK/MAPK and PI3K/AKT signaling pathways and inhibiting the proliferation of tumor cells (114, 115). Here, by this case, we have proven that pyrotinib is efficient in HER2-positive LM, although its efficiency might have partly owed to its combinational application with other drugs. But poor compliance of the patient with intrathecal methotrexate led to irregular and insufficient intrathecal administration and discontinuation of methotrexate before disease progression. In addition, leukodystrophy was also tracked in MRI. Therefore, intrathecal methotrexate may play a limited role in the treatment of the patient.

Furthermore, metronomic chemotherapy was a feasible treatment of advanced cancer, particularly those in poor physical conditions, such as the elderly (116). The effectiveness and safety of oral vinorelbine metronomic chemotherapy in treating elderly MBC patients have since been established by a phase II clinical investigation which found a mPFS of 7.7 months and a mOS of 15.9 months in the entire population of patients, comparable to traditional chemotherapy findings. There was no drug reduction or withdrawal and no grade 3-4 adverse events occurred. Besides, the VICTOR-2 study demonstrated that the metronomic vinorelbine plus capecitabine was curative for MBC with a low incidence of grade 3-4 adverse events and no incidents impairing patients’ quality of life (117). For that reason, in the beginning, when our patient could not tolerate traditional chemotherapy, we advised the patient to try metronomic chemotherapy. Also, a study by Wildiers H et al. showed that there was a difference of 7-months in the mPFS between the two evaluated groups (12.7 months for trastuzumab and pertuzumab plus metronomic oral cyclophosphamide *vs*. 5.6 months for trastuzumab plus pertuzumab, *p* = .12), highlighting the advantages of combining metronomic chemotherapy with targeted therapy to treat HER2-positive MBC and giving credibility to our choice of treatment (118).

There are several limitations of the present study that should be addressed. Firstly, the literature review for LM in HER2-positive BC found little findings in observational studies and clinical trials, focusing instead on case reports or case series, which were truly subject to the current state of research. Secondly, the literature research was performed by “keywords”, which may lead to inevitable omissions and it was difficult to describe all information for each study. Thirdly, given the present complete-matching literature, we were unable to draw a firm conclusion for optimal treatment regimen in HER2-positive BC with LM from nonrandomized controlled studies or case report carefully selected by clinicians with selection bias.

Encouragingly, the patient responded significantly with the combination of pyrotinib-targeted therapy and metronomic vinorelbine, with a 16- month survival time and enhanced quality of life. As far as we know, the survival time of the patient has exceeded the current average level, as reported in the past literature (119). Besides, this tailored treatment regimen was quite well-tolerated, and efficiently relieved symptoms of the patient caused by leptomeningeal disease, which exceeded our expectations.

## Conclusion

The treatment for HER2-positive BC with LM is still at the exploratory phase, though, with no revolutionary breakthrough in recent years. Clinical trials focused on many novel drugs such as T-DM1, DS8201, tucatinib, and even immunotherapy have been conducted extensively. This case is merely a one-person report but revealed that pyrotinib plus metronomic vinorelbine was effective and tolerable in HER2-positive BC with LM. Based on multidisciplinary cooperation to develop the final treatment regimen, we constituted a successful step forward. Therefore, the treatment strategy of pyrotinib with metronomic vinorelbine is worthy to be further investigated in large-scale clinical trials for HER2-positive BC patients with LM to improve the prognosis of this population.

## Author Contributions

Conceptualization: YC and HL. Treatment decision-making and discussions: MS, LX, HG, RT, LS, BZ, BC, and HL. Data collection and analysis: YC and SY. Manuscript writing: YC. Final approval of manuscript: HL. All authors contributed to the article and approved the submitted version.

## Funding

We also would like to acknowledge the funding support of the National Natural Science Foundation of China (Grant No. 81902713), the Breast Disease Research Foundation of Shandong Provincial Medical Association (Grant No. YXH2020ZX066) and the Chinese Society of Clinical Oncology-Heng Rui Cancer Research Foundation (Grant No. Y-HR2019-0432; Y-HR2018-121).

## Conflict of Interest

The authors declare that the research was conducted in the absence of any commercial or financial relationships that could be construed as a potential conflict of interest.

## Publisher’s Note

All claims expressed in this article are solely those of the authors and do not necessarily represent those of their affiliated organizations, or those of the publisher, the editors and the reviewers. Any product that may be evaluated in this article, or claim that may be made by its manufacturer, is not guaranteed or endorsed by the publisher.
